# Cariporide and other new and powerful NHE1 inhibitors as potentially selective anticancer drugs – an integral molecular/biochemical/metabolic/clinical approach after one hundred years of cancer research

**DOI:** 10.1186/1479-5876-11-282

**Published:** 2013-11-06

**Authors:** Salvador Harguindey, Jose Luis Arranz, Julian David Polo Orozco, Cyril Rauch, Stefano Fais, Rosa Angela Cardone, Stephan J Reshkin

**Affiliations:** 1 Instituto de Biología Clínica y Metabolismo (IBCM), Postas 13–01004, Vitoria, Spain; 2 School of Veterinary Medicine & Science, University of Nottingham, Sutton Bonington Campus, LE12 5RD, Sutton Bonington, UK; 3 Dipartimento del Farmaco, Istituto Superiore di Sanità, Rome, Italy; 4 Department of Therapeutic Research and Medicines Evaluation, National Institute of Health, Rome, Italy; 5 Department of Bioscience, Biotechnology and Biopharmaceutics, University of Bari, 70126, Bari, Italy

**Keywords:** pH and cancer, Cancer etiopathogenesis, Proton transporters, Proton transport inhibitors, Cariporide in cancer, NHE1 and cancer, Cancer treatment, Multiple drug resistance, New therapeutic paradigm in cancer

## Abstract

In recent years an increasing number of publications have emphasized the growing importance of hydrogen ion dynamics in modern cancer research, from etiopathogenesis and treatment. A proton [H^+^]-related mechanism underlying the initiation and progression of the neoplastic process has been recently described by different research groups as a new paradigm in which all cancer cells and tissues, regardless of their origin and genetic background, have a pivotal energetic and homeostatic disturbance of their metabolism that is completely different from all normal tissues: an aberrant regulation of hydrogen ion dynamics leading to a reversal of the pH gradient in cancer cells and tissues (↑pH_i_/↓pH_e_, or “proton reversal”). Tumor cells survive their hostile microenvironment due to membrane-bound proton pumps and transporters, and their main defensive strategy is to never allow internal acidification because that could lead to their death through apoptosis. In this context, one of the primary and best studied regulators of both pH_i_ and pH_e_ in tumors is the Na^+^/H^+^ exchanger isoform 1 (NHE1). An elevated NHE1 activity can be correlated with both an increase in cell pH and a decrease in the extracellular pH of tumors, and such proton reversal is associated with the origin, local growth, activation and further progression of the metastatic process. Consequently, NHE1 pharmaceutical inhibition by new and potent NHE1 inhibitors represents a potential and highly selective target in anticancer therapy. Cariporide, being one of the better studied specific and powerful NHE1 inhibitors, has proven to be well tolerated by humans in the cardiological context, however some side-effects, mainly related to drug accumulation and cerebrovascular complications were reported. Thus, cariporide could become a new, slightly toxic and effective anticancer agent in different human malignancies.

## Rationale

### Proton transport and its inhibition as an approach to cancer etiopathogenesis and treatment

The pathological regulation of hydrogen ion dynamics in cancer cells and tissues leads to a reversed hydrogen ion gradient in these cells (↑pH_i_/↓pH_e_). This results in a very acidic extracellular microenvironment specific to all malignant tumors [1-5]. Thus, malignant cells have an acid–base balance that is completely different to that observed in normal tissues and that increases with increasing neoplastic state: an extracellular acid microenvironment (pH_e_) linked to a 'malignant’ alkaline intracellular pH (pH_i_) [4]. Indeed, tumor cells have alkaline pH_i_ values of 7.12-7.7 vs 6.99-7.05 in normal cells while producing acidic pH_e_ values of 6.2-6.9 vs 7.3-7.4 in normal cells (Table 1) [4,6,7]. This specific and pathological reversal of the pH gradient in cancer cells and tissues compared to normal tissues (“proton reversal”) is now considered to be one of the main characteristics defining tumor cells that completely alters their thermodynamic balance and molecular energetics, regardless of their pathology and genetic origins. The induction and/or maintenance of intracellular alkalinization and its subsequent extracellular/interstitial acidosis on intratumoral dynamics have been repeatedly implicated as playing an essential, direct and pivotal role both in cell transformation and growth as well as in the active progression and maintenance of the neoplastic process [8-10]. Moreover, this intracellular alkalosis represents a common final pathway in cell transformation through the stimulation of the Na^+^/H^+^ transporter by a myriad of carcinogens of the most varied origins and natures [8-19]. Such a wide array of carcinogens induce cell transformation and an increase in cell pH [14,17-24].

**Table 1:** **pH**_**i **_**and pH**_**e **_**in normal and cancer cells: apoptosis and antiapoptosis**

	**Normal cells**	**Cancer cells**
	**(**pH_**i **_**<** pH_**e**_)	**(**pH_**i **_**>** pH_**e**_)
		***(“proton gradient reversal”)***
**Intracellular pH (pH**_**i**_**)**	**6.99-7.05**	**7.12-7.7**
		**(Pathological antiapoptosis)**
**Extracellular/**		
**interstitial pH (pH**_**e**_**)**	**7.35-7.45**	**6.2-6.9**
**pH**_**i **_**<6-6.5**		**(Therapeutic apoptosis)**

For further details, see text and refs. [4,40].

### Factors that increase cell pH and/or stimulate NHE activity as mediators of high pH_i_-mediated carcinogenicity

Virus (HPV E5, human polioma virus)

Oncogenes and viral proteins (v-mos, Ha-Ras, HPV16 E7)

Gen products (Bcl-2)

p53 deficiency

Chemicals carcinogens (arsenic salts, etc.)

Chronic hypoxia and HIF

Hormones (insulin, somatostatin, growth hormone, glucocorticoids)

Growth factors (IGF-1, HGH, PDGF, VEGF, EGF, IL-1, IL-8, TGF-β, G-CSF,

Angiotensin II, PGE_2_, diferric transferrin, bombesin)

Such an elevated pH_i_ was very early on implicated as a crucial factor and target in neoplastic transformation in response to the overexpression of certain proton transporters as well as the ras and v-mos oncogenes [25,26]. It was observed that oncogene-dependent transformation resulted in an elevated pH_i_, increased NHE1 activity and increased glycolysis, although it was not clear from those early experiments if the driving factor was the stimulated NHE1, an elevation of pH_i_ or the increased glycolysis itself. This question was resolved in a study utilizing the inducible expression of an oncogene (HPV16 E7) to dissect the time-dependence of the appearance of the three above-mentioned factors [10]. This study demonstrated that the first step in oncogene-dependent transformation of normal cells is the activation of the NHE1 with the subsequent cytosolic alkalinization followed by an increase in glycolysis. Furthermore, it was demonstrated that this alkalinization was the driver of a series of transformation hallmarks such as increased growth rate, substrate-independent growth, growth factor independence and tumor growth [4,18,27,28]. Altogether, these data demonstrate that oncogenes utilize NHE1-induced cellular alkalinization to produce the unique cancer specific pH regulation with the resulting pH-related hallmark phenotypes characteristic of cancer cells. NHE1, by controlling pH_i_ and preventing cell acidification plays a key role in cell survival/proliferation and tumour growth. Even from an epidemiological perspective, it was recently shown that low concentrations of arsenic salts in drinking water induce a carcinogenic effect directly related to the onset of different human tumors and that this effect is mediated by the stimulation of NHE1 and the resulting increase in cytosolic pH. These authors concluded that the increase in cell pH is an important pathogenetic mediator of the carcinogenic effects of arsenic salts [14], as has been reported in other parallel studies by different groups of researchers [4,8-10,13,25,28]. This is in line with previous reviews reporting a cause-effect relationship of a high microenvironmental pH and/or NHE stimulation with both pH-directly and pH-indirectly carcinogenesis, with the effects of a high pH reproducing most of the characteristics and metabolic behaviour of cancer cells [11].

Importantly, these complex dynamics of pH-metabolism engage the cell in a vicious cycle from very early on: the oncogene-driven alkalinization increases glycolysis and proliferation which, by generating a need for a high energy consumption, creates a high proton production that activates various proton efflux transport systems resulting in a further alkalinisation of the cell. This even further reduces oxidative phosporylation (OXPHOS) and increases glycolysis. This “chain-reaction” of deep-seated and dynamically disregulated H^+^ energetics creates a “perfect storm for cancer progression” [2]. Finally, to our knowledge the pathological alkaline pH_i_ of tumor cells and tissues have never been described in any other type of cell or disease other than malignancy [29,30]. This adds further weight to the paradigm concerning the specificity and selectivity of these H^+^-mediated, deep-seated energetic abnormalities regarding the advantageous thermodynamics of the malignant process.

**Figure 1:**
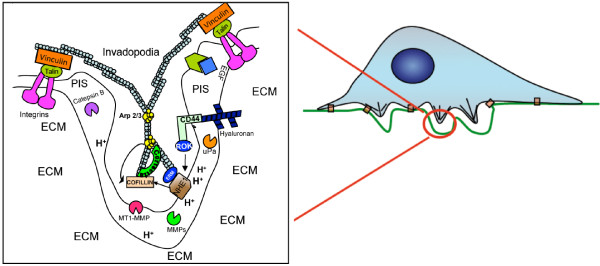
**Model of localization and role of NHE1 in invadopodia.** The insert is a magnification of the F-actin-enriched cellular protrusions into the ECM that are responsible for ECM degradation and are known as invadopodia. Invadopodia formation is activated by integrin binding to the ECM and their activity further increased through the CD44 (activated by its ligand Hyaluronan) and EGFR receptors located in the membrane. The integrin receptors are connected to the cytoskeleton (blue circles) through the proteins Talin and Vinculin. The proteases cathepsin B, D and L, Urokinase Plasminogen Activator and the matrix metalloproteinases MMP-2 and MMP-9 are released extracellularly while the MT1-MMP is localized within the membrane and participates together with Cathepsin B in the processing of inactive pro-MMP-2 into active MMP-2. Glycolytic enzymes are enriched in invadopodia, leading to the localized production of protons. These protons are secreted via an active NHE1 that is recruited to the invadopodia through integrin binding and further stimulated by CD44 and EGFR. NHE1 with its two functions (scaffolding protein and ion exchanger) leads to membrane protrusion and proteolysis. As a proton transporter, NHE1 promotes invasion through its control of the acidification of the peri-invadopodial space where NHE1 proton secreting activity and proteases act in concert to degrade the ECM during invasion. Further, the NHE1-dependent alkalinization of the invadopodia cytosol results in a phosphorylation of cortactin with the subsequent release of cofilin which promotes actin polymerization, growth of the invadopodia cytoskeleton and invadopodia protrusion. Secondly, NHE1 also promotes invadopodial formation via its interaction with the cytoskeleton through its binding to the actin anchoring protein, ezrin, which, reciprocally is responsible for the localization of NHE1 to the invadopodia in response to ECM and growth factor receptor activation. PIS: PeriInvadopodia Space; ECM: ExtraCellular Matrix. Please see text for discussion and references.

Indeed, this “basic” and specific abnormality of the relationship between the intracellular and the extracellular proton dynamics (“proton gradient reversal”) represents a phenomenon that is increasingly considered to be one of the most differential hallmarks of cancer [4,5]. This severe abnormality in cell physiology has led to the formation of a unifying thermodynamic view of malignancy, a comprehensive new paradigm able to encompass an enormous and scattered bulk of information in the main areas of research that embraces many different and so far poorly interconnected cancer fields. These range from etiopathogenesis, cancer cell metabolism, multiple drug resistance (MDR), neovascularization and the metastatic process to selective apoptosis, cancer chemotherapy, cancer epidemiology and even the, so far poorly understood, phenomenon of the spontaneous regression of cancer [27,31,32]. Further, the increased diffusion of the proton ions along concentration gradients from tumors into adjacent normal tissues creates a pericellular and peritumoral acidic microenvironment involved in driving destruction of the surrounding normal limitrophic tissue, invasion and metastasis. Both the acidic pH_e_ and the constitutively active NHE1 play a key role in driving protease-mediated digestion and remodelling of the ECM and the turning on of invasive phenotypes of the cell, scavenging normal tissue and increasing motility through the formation of invasive structures such as leading-edge pseudopodia and invadopodia [4,6,33-35] (Figure 1). Furthermore, focal cell to cell adhesions are particularly located at the cell front where NHE1 is concentrated. These sites feature a remarkably alkaline cytosolic and an acidic pericellular pH and thus a much steeper proton gradient across the plasma membrane compared to the rest of the cell [36]. Most recently it has also been advanced that that deregulation of NHE1 activity is a major factor leading to metastasis in human breast cancer [37]. Altogether, this clearly indicates that therapeutic targeting of the main proton transporters that are selectively overexpressed in cancer cells could be highly specific for malignancy, and is likely to open new pathways towards the development of more effective and less toxic chemotherapeutic measures for all solid malignant tumors and leukaemias [5,38-40].

### The pH of cancer cells and the Warburg Effect: a synthetic explanation

Even from the time of Warburg´s death in 1970 the idea that the shift to glycolytic metabolism relative to OXPHOS under aerobic conditions could be explained by an increase in the intracellular pH, has been increasingly gaining weight with the passing of time [8,28,41-44]. Nagata et al., have recently reached a synthetic conclusion: that the Warburg effect may be simply, and perhaps fully explained by the elevation of pH_i_ in cancer cells [44]. These groups have also shown that malignant alkalinisation drives the initial activation of aerobic glycolysis (first appearance of the Warburg Effect) [44,45]. In the presence of adequate oxygen levels, the intracellular pH plays a key role in determining the way cancer cells obtain energy: an alkaline pH_i_ driving aerobic glycolysis and an acidic pH driving oxidative phosphorylation [28]. An explanation for this phenomenon derives from the fact that both the processes of OXPHOS and glycolysis are exquisitely but oppositely pH sensitive and a rapid shift of cell metabolic patterns follows either acidification or alkalinisation. On the one hand, it has been known for decades that an alkaline pH_i_ even slightly above steady-state levels stimulates the activity of key glycolytic enzymes such as phosphofructokinase (PFK-1) and inhibits gluconeogenesis [8,41,42,46,47]. Indeed, in cancer cells a high pH_i_ situation can increase the allosteric regulation of PFK-1 more than 100-fold [2,8]. This also has important diagnostic consequences. We know that the selectivity of PET technology is based upon the degree of tumor glycolysis. Tumoral glycolysis is to a great extent dependent on pH_i_, increasing with cellular alkalinity and decreasing with intracellular acidification. This feature opens a diagnostic potential for the development of new radiological methodologies based on the determination of the intracellular acid–base status. Thus, measurements of the pH_i_ in malignant tumors, and even premalignant conditions, could become a better diagnostic tool than PET technology in determining the presence of a tumor, and could also detect higher than normal pH_i_ areas (> 7.2/7.3) where malignancy is most likely to develop and eventually manifest itself [35,48-51]. Finally, hypoxia (low pO_2_) or alkalosis (high pH_i_ or low intracellular H^+^-concentration) show similar effects on cell intermediary metabolism as well as a parallel transforming potential [11]. Indeed, the transforming effects of a high pH_i_ are known as “para-hypoxia” and the carcinogenic effects of hypoxia as the “Warburg-Goldblatt effect”, after Goldblatt induced malignant transformation of cells kept in relatively low O_2_, non-killing conditions during Warburg´s time (Table 2) [11,52]. Indeed, it can now be considered that the high pH_i_ of tumor cells, the Warburg effect and the steady-state cancer cell proton reversal may very well represent one and the same phenomenon observed from different perspectives, at different historical times and through less outreaching and integral perspectives.

**Table 2:** **Similarities of effects of a high pH - Low [H**^**+**^**] or (Alkalosis) and low pO**_**2 **_**(Hypoxia) on cellular biochemistry and metabolism**

	**Hypoxia**	**Alkalosis**
**Glycolysis**	**↑**	**↑**
**Phosphofructokinase**	**↑**	**↑**
**Pyruvate production**	**↑**	**↑**
**Lactate production**	**↑**	**↑**
	**(Anaerobic glycolysis)**	**(Aerobic glycolysis)**
**ATP production**	↓	↓
Mitochondrial oxidation	↓	↓
Transforming/oncogenic effect	**+**	+
	**(Goldblatt-Warburg effect)**	**(The Warburg effect)**

↑ Stimulation; ↓ Inhibition.

For further details, see text and reference [11].

Note: Reprinted from Critical Reviews Oncogenesis, vol. 6. Harguindey S, Pedraz JL, Garcia Canero R, Perez de Diego J, Cragoe EJ, Jr. Hydrogen ion dependent oncogenesis and parallel new avenues to cancer prevention and treatment using a H(+)-mediated unifying approach: pH-related and pH-unrelated mechanisms, p. 6, **©**1995, with the permission from Begell House, Inc.

### Back to beginnings: a fatal historical error?

To understand the full significance of the most recent observations and data we need to go back in time to the beginnings of cancer biochemistry, and so, to the postulated origin of cancer cells [53,54]. By doing so we realize that a fundamental confusion in the entire field of metabolic and biochemical cancer research was created from its very beginning. Nowadays, it is clear that Otto Warburg was wrong on the main point of his famous theory, namely, on the levels of cancer cell pH_i_, and consequently on its relationship to glycolysis*.* Indeed, Warburg believed that the pH of cancer cells was acid because of their high production rates of lactic acid [55-57]. Probably, the main reason for overlooking the true pH/glycolysis relationship, or at least for being given a secondary role at that time was that, during the 60’s and 70’s, the necessary technology to measure pH_i_ was not available [58]. The situation started to turn around just after Warburg’s death in 1970, when different reports began to emphasize that the pH_i_ of cancer cells was the opposite from what was generally thought during Warburg’s life [18,41,43,58]. Thus, Warburg could not have been aware that cellular alkalosis not only activates glycolysis but at the same time hinders oxidative phosphorylation and the entrance of pyruvate in the Krebs cycle [42,59]. This allows a further insight into the reasons behind decades of confusion and disagreements on his theory of “the abnormal respiratory mechanisms of cancer cells”, that he defended all his life [8,28,42,53,59-61]. It is also important to remember that at Warburg’s time there were not techniques permitting the discrimination between the pH of the cytosol and of the internal organelles. Today we are able to show that within tumor cells the cytosol is alkaline while the cytoplasmic vesicles are very acidic [62,63]. This is possible thanks to proton pumps, on one side eliminating protons outside the tumor cell when expressed on the plasma membrane, while pumping them from the cytosol to the internal lumen of the acidic vacuoles in order to avoid internal acidification (reviewed in [64]).

Importantly, any consideration concerning the intimate relationship of high pH_i_ and glycolysis was fully missed during the famous arguments between Warburg and Weinhouse published in Science in 1956 [61,62]. Indeed, all those heated discussions could only beg the real issue and could have been obviated if the true effect of pH on anaerobic and aerobic glycolysis and oxidative phosphorylation (“parahypoxia”) [11] could have been taken into account. Probably, this is also the main reason behind the fact that the search for the real cause underlying the Warburg effect has created many disagreements over the last decades [3,56,61,63-71]. All in all, it can now be said that Warburg was right up to a certain point but that his critics were also partially right. However, all of them missed the main point. Aerobic glycolysis or damaged respiration was not the primary cause of cancer, as Warburg defended until his death. Indeed, the primary cause of cancer appears to be, precisely, the main cause of the aerobic glycolysis of tumors: a profound disruption of the homeostatic acid-balance of the cell mainly represented by an abnormally high pH_i_ mediated by an extremely varied number of etiological factors of different natures. In summary, cellular alkalosis represents a common final pathway in cell transformation induced by a myriad of different stimuli, from oncogenes to virus to mitogens to growth factors and hormones to gene products [1,4,8-10,27]. Finally, some recent and otherwise complete reviews dealing with Warburg’s contributions to modern concepts in cancer metabolism, tumor glycolysis, the initiation of cancer and oxidative phosphorylation have not considered the tight cause-effect interrelationships between pH and glycolysis, the Warburg effect and cancer proton reversal [65,68,69,72,73].

### Anticancer potential of NHE inhibitors. Background to recent developments

**Figure 2:**
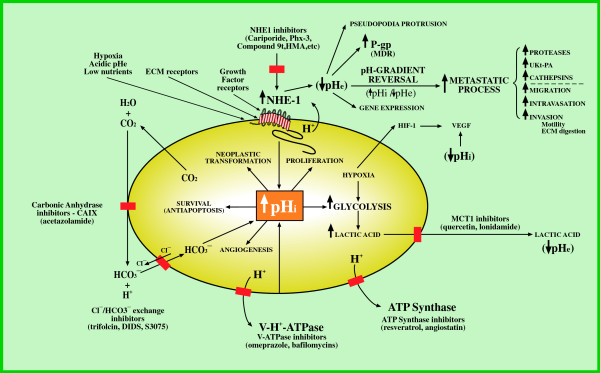
**Dysregulated pH-control systems in cancer cells.** Targets for proton transport inhibitors (PTIs) as anticancer agents. Nos. 1, 2, 3, 4, 5 and 6: Mechanisms that induce intracellular alkalinisation as the key factor in cell transformation and progression with its secondary abnormalities. Secondary pH_i_-dependent extracellular acidification, pH-gradient reversal and hypoxia as triggers for the metastatic process. Five targets for inhibition of proton extrusion of cancer cells as targets for metabolically-directed anticancer treatment and examples of drugs of the different proton transport inhibitors at the sites of their activity. For further details see text and ref. [5] Abbreviations: NHE1: Na^+^/H^+^ exchanger: HMA; 5-(*N,N-*hexamethylene)-amiloride; Phx-3: 2-aminophenoxazine-3-one; Compound 9 t: 5-aryl-4-(4-(5-methyl-1*H*-imidazol-4-yl) piperididn-1-yl)pyrimidine analog; HIF-1: hypoxia-inducible factor; MCT1: monocarboxylate transporter or H^+^-lactate co-transporter; CAIX: carbonic anhydrase IX; V-H^+^-ATPase: vacuolar H^+^-ATPase; VEGF: vasoendothelial growth factor; UKT-PA: urokinase-type plasminogen activator; P-gp: P-glycoprotein; MDR: multiple drug resistance; pH_i_: intracellular pH; pH_e_: extracellular/interstitial tumoral pH.

The development and maintenance of this reversed pH gradient is directly due to the ability of the tumor cells to secrete protons (H^+^) [1,4,27,74]. This proton secretion depends on the buffering capacity of the cell and is driven by a series of membrane-bound proton transporters (MBPT), mainly the Na^+^/H^+^ exchangers but also carbonic anhydrases (CAs, mainly CA IX and XII), vacuolar H^+^-ATPases, the H^+^/Cl^-^ symporter, the monocarboxylate transporter (MCT, mainly MCT1), also known as the lactate-proton symporter, the Na^+^-dependent Cl^-^/HCO_3_^-^ exchangers and ATP synthase [1,5,40,74-77], each of them having its specific inhibitors (Figure 2). The human NHE (SLC9) family is comprised of nine A isoforms (SLC9A1-9) with one established (NHE6) and one possible (NHE1) splice variant and five pseudogenes plus two B isoforms (SLC9B1/2) and two C isoforms (SLC9C1/2) [78]. (For a more detailed information about the SLC gene tables, please visit: http://www.bioparadigms.org).

Among them, the most important, functionally active, cancer-selective and better studied is the Na^+^/H^+^ exchanger isoform one, NHE1 [79-81]. The NHE1 is specifically involved in cellular acid–base balance and is the predominant isoform expressed in tumors, where it has been shown that it contributes to cellular pH homeostasis, cell transformation, proliferation, motility, migration, tumor growth, invasion, activation of the metastatic process, resistance to chemotherapy and probably also to the spontaneous regression of cancer [4,31,37,82-84]. Conversely, decreasing NHE1 expression or inhibiting NHE1 activity leads to tumour cell growth arrest, inhibition of glycolysis, acidification of the intracellular space and selective apoptosis [29,38,45,82]. An elevated NHE1 activity is considered to be the major factor in promoting tumor extracellular/interstitial acidity from even the earliest pre-cancer stage of oncogene-driven neoplastic transformation [25,26]. However, large studies of patient cohort samples demonstrating that NHE1 is overexpressed in human tumors are lacking. Also, some cancer cells can be NHE1 negative and maintain cytosolic alkalinisation through expression of other MBPT [40,85]. Consequently, NHE1 inhibitors appear predestined to be taken advantage of as a therapeutic target in probably most types of human cancer [81,86-89]. For a detailed review of the structure and biophysical characteristics of NHE1, the regulation of the NHE1 activity and its role in tumor cells pH homeostasis, please refer to recent publications [2-4,79,80,90].

Beyond studying in depth the evolution and progress of biochemical and metabolic cancer research, a major purpose of this review is to consider the fact that the new and selective NHE1 inhibitors show promise to become potent anticancer agents in preclinical trials and, eventually, in cancer patients. Amiloride was the first NHE inhibitor developed and it was shown to decrease vasoendothelial growth factor (VEGF) production and the activity of urokinase-type plasminogen activator (μPA), metalloproteinases (MMP) and other proteases, all of which aid in the activation of the metastatic process [89,91-94]. Amiloride alone was shown to achieve a complete *in vivo* anti-metastatic effect in transplanted tumors in rats [95]. Indeed, there are occasional reports of long-term treatment with amiloride in humans achieving remissions of cancer after chemotherapy had failed to control disease progression [96]. Recent publications on the use of amiloride in cancer therapy discussed the different studies where its use had clear anti-neoplastic effects with few side-effects [97]. Long before this, the utilization of amiloride and its derivatives were proposed as anticancer agents in bedside oncology [86]. This potassium-sparing diuretic, apart from having a direct antitumoral, antimetastatic and antiangiogenic effect [95,97,98], at least in part by inhibiting uPA and VEGF, has been shown to be well tolerated and safe when used in the chronic situation in pharmacological dosages in humans, the main side-effect being occasionally increased plasma K^+^ levels [96,99,100]. Since more selective and powerful NHE inhibitors, like cariporide, are not available for human use, amiloride should still be part of new protocols dealing with the concerted use of a cocktail of proton transport inhibitors (PTIs) in different human solid tumors [5,96,101].

For many years investigators have waited for more specific and potent NHE inhibitors to be developed and be made available to the clinician [102]. In this vein, powerful amiloride analogues, like ethylisopropylamiloride (EIPA), have been studied in different settings regarding its anticancer potential [103-105]. Hexamethylamiloride (HMA) and dimethylamiloride (DMA), were also introduced in basic experimental research and provided additional evidence of the validity of this approach. Striking results in different kinds of leukemic cells were reported with the potent NHE1 inhibitor HMA, which specifically decreases the pH_i_ well below the survival threshold leading to selective apoptosis in a variety of human leukemic cells [38]. This has led to the consideration that inducing a low pH_i_-mediated apoptosis as a cancer-specific therapeutic modality for all cancer cells and tissues could be a new and original approach to clinical therapeutics [27,39,44,76,106]. Regarding NHE-related malignant angiogenesis, the activity of a significant number of proangiogenic factors and oncogenes has been shown to positively affect NHE1 expression while, on the contrary, a wide array of anti-angiogenic drugs inhibit NHE1 [107,108]. In summary, a great deal of evidence has been accumulating showing that the NHE is an important, and possibly selective, anticancer target [11,81,86,87,89,100]. The pharmacology and therapeutic possibilities of the rest of the different proton transporters besides NHE1 have been thoroughly reviewed recently and will not be further dealt with here [4,75,81,83].

### Cariporide’s anticancer potential

It has been demonstrated that treating various kinds of cancer cells with selective and potent inhibitors of NHE1, including cariporide, suppresses their invasive capability [37,109-111].

Di Sario *et al.,* have also shown that cariporide, through its selective inhibition of NHE1 and subsequent decrease of intracellular pH reduces proliferation and induces apoptosis in cholangiocarcinoma cells [112], leading these authors to suggest the potential therapeutic value of cariporide against this human tumor. A recent review has also focused on how to therapeutically target the NHE1-mediated metabolic transformations of cancer cells with cariporide [64]. However, translation to the oncology clinic has yet to be realized because, unfortunately, the utilization of this drug in cancer treatment has not been explored [4,84] and there is scarce data on NHE1 upregulation in tumour cells [40]. This is most important since the concerted utilization of less potent and specific inhibitors of NHE1 and other proton transport inhibitors (PTIs) was recently advanced as a new, selective and integrated anticancer strategy [5,101] (Figure 2).

The only non-amiloride based compounds with NHE1 inhibitory activity that have undergone clinical trials are cariporide and eniporide, and, unfortunately, those trials were not in the field of cancer but in a cardiological setting and for ischaemic-reperfusion injury. An early study on the effect of cariporide in 100 patients waiting to receive perfusion therapy via primary coronary angioplasty within 6 hours of the onset of symptoms suggested that reperfusion injury could be a target for NHE inhibitors and these results led to further clinical trials to confirm the therapeutic potential of NHE inhibitors [113]. Two were with cariporide: The “Guard During Ischemia Against Necrosis” (Guardian) [114,115] and “The Na^+^/H^+^ Exchanger Inhibition to Prevent Coronary Events in Acute Cardiac Conditions” (EXPEDITION) [116]. The “Guardian” trial included a total of 11590 patients with unstable angina or a myocardial infarction who received placebo or different doses (30, 80 and 120 mg) of cariporide. There were an early clinical benefit and elevated six month survival rate in only a group of patients requiring urgent coronary bypass graft surgery and at a cariporide level of 120 mg. There was also a trial utilizing eniporide: “The Evaluation of the Safety and Cardioprotective Effects of Eniporide in Myocardial Infarction” (ESCAMI) [117].

Despite the cardioprotective value of cariporide in reducing myocardial infarcts in both the EXPEDITION and in the earlier GUARDIAN trials, use of the drug was associated in the EXPEDITION study with a significant increase in the rate of mortality (from 1.5% to 2.2% at day 5) due to an increase in cerebrovascular events [116,118]. The appearance of these adverse effects in the last trial can probably be ascribed to the higher cumulating dose of cariporide administered in the EXPEDITION trial with respect to the GUARDIAN trial [119].

Clearly, a clinically reasonable initial approach in an oncology setting would be to minimize the systemic dose of the drug in order to dissociate the adverse and probably off-targets effects from the beneficial effects. Interestingly, rats having a lifelong treatment with cariporide had a greatly extended lifespan and this was interpreted as being due to a reduced occurrence of cancer [120]. Finally, cariporide has been shown to be useful in overcoming multiple drug resistance (MDR) and the activity of the metastatic process [121]*.* Besides, it is orally bioavailable and by this route of administration has been used but, unfortunately, never to date as an anticancer drug [114-119,122-125]. Interestingly, hypoxia is part of the tumor metabolic microenvironment and has been shown to activate NHE1 and consequent invasion [109,126,127]. Cariporide reduces hypoxia-mediated tumor invasion of human tongue squamous cell carcinoma by inhibiting NHE1 [128]. In this study, the authors demonstrated that inhibition of NHE1 by cariporide (HOE642) suppressed the invasion and migration of Tca8113 cells under hypoxic conditions. In another study pharmacological inhibition of p38 MAPK (mitogen-activated protein kinase) also significantly suppressed C/EBPα expression under hypoxia conditions after NHE1 inhibition [110]. These results indicate the enhancement of hypoxia-induced K562 differentiation by NHE1 inhibition, which may be due to up-regulation of C/EBPα via p38 MAPK signalling pathway, which suggests a possible therapeutic target of NHE1 under hypoxia microenvironment in the treatment of leukaemic diseases. Finally, this also suggests that NHE1 inhibitors could be combined in clinical trials with antiangiogenics [129,130] because tumor hypoxia and/or acidosis also stimulates VEGF [131,132].

Indeed, in addition to VEGF release and, subsequently, neoangiogenesis, being stimulated by hypoxia, upregulation of VEGF has also been linked as being secondary to acidic pH_e_[131,133]. Also, NHE1-dependent lowering in pH_i_ also reduces the release of VEGF from the tumor cell so hindering motility and invasion [38,134]. Systemic amiloride treatment also reduced experimentally-induced neovascularization in an animal model; probably through inhibition of NHE1 [135]. For more detailed information please refer to the following review [107]. Importantly, the potency of cariporide and some other NHE1 inhibitors is related to the ionization state of the guanidine residues. In this respect, the acidic extracellular pH of tumors (which can be as low as 6.2) will render zoniporide (pK_a_ = 7.2), TY-12533 (pK_a_ = 6.93) and, especially, cariporide (pK_a_ = 6.28) positively charged. Therefore, the acidic tumor microenvironment could turn out to be an advantage in terms of dose-dependent side-effects as these compounds would be more efficient at inhibiting NHE1. Indeed, cariporide would be even more active at a very low pH_e_ (ie. IC_50_ = 22 nM vs 120 nM at pH_e_ 6.2 and 6.7, respectively) [136-138]. Finally, the development of new non-guanidine derived NHE1 inhibitors could alleviate some of the detrimental side-effects found in the Expedition trial (see section below on the new and potent non amiloride-derived and non guanidine-derived compounds).

### The role of pH in multiple drug resistance (MDR)

#### *Cariporide and other proton transport inhibitors in the overcoming of MDR*

**Figure 3:**
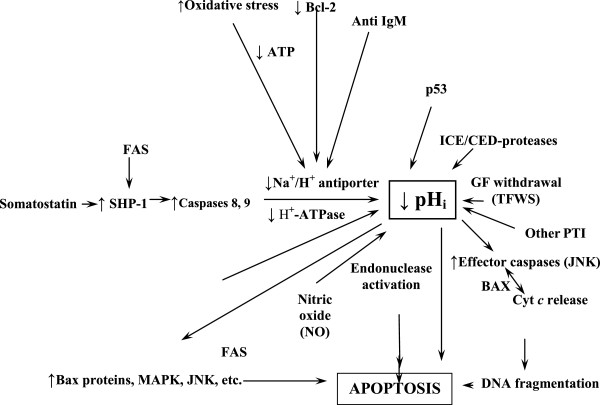
**Intracellular signaling factors and mechanisms targeting pH**_**i **_**and the Na**^**+**^**/H**^**+ **^**exchanger in the apoptosis of cancer cells.** This integrated and homeostatic pH-related perspective can help to foretell pro-apoptotic and anti-apoptotic factors in order to find synergistic therapies and potential antagonisms (MDR) in anticancer treatment. Abbreviations: ↑: Stimulation; ↓: inhibition; SST: somatostatin; SHP1: protein tyrosine phosphatase; MDR: multiple drug resistance; GFs: growth factors; Cyt C: cytochrome C; NO: nitric oxide. TFWS: trophic factor withdrawal syndrome; αCD95 (Fas/Apo-1) death receptor; JNK: Jun-terminal kinase; MAPK: mitogen-activated protein kinase; PTI: proton transport inhibitors; ICE: interleukin-1β-converting enzyme. (For further details, see text and refs. [5,29,30]. (Modified from refs. [5,29] by permission from Dove Medical Press, Ltd., and Anticancer Research).

**pH, MDR and cancer** A direct cause-effect relationship among MDR and the elevation of pH_i_ in cancer has been recognized by different groups of researchers [83,139-141]. On the contrary, the failure of tumor cells to die following chemotherapeutic treatment appears to be highly dependent on their resistance to undergo intracellular acidification, a situation that is apparently necessary as a prior and early condition that allows cancer cells to engage in a tumor-specific apoptotic process [38,44,45,106,112,142] (Figure 3). Cancer cells are known to establish a dynamic and well organized self-defensive anti-apoptotic strategy (*“the neostrategy of cancer cells and tissues*”) [27] which is mediated through different anti-acidifying mechanisms such as hyperactivity of the group of membrane-bound proton extrusion transporters, inactivation of Bcl-2, Bcl-_xl_ and/or a pH-dependent de-stabilization of p53 [12,13,139]. These concerted dynamic changes work as an anti-chemotherapeutic shield involved in multiple drug resistance (MDR) and in the development of newly resistant subpopulations of tumor cells [1,143]. The final therapeutic aim is to target this selective acid–base disruption of cancer cell metabolism based on the H^+^-dependent thermodynamic advantages that malignant cells possess for their evolutionary survival as compared to their normal counterparts in order to exploit such differences in selective cancer therapeutics [144-146]. This can be achieved with the concerted utilization of proton transport inhibitors (PTIs) as primary treatment and also as an adjuvant measure in overcoming MDR, increasing therapeutic specificity and effectiveness regardless of tumor type and origin [4,82,83]. Besides, drugs of the amiloride series and/or other proton transport inhibitors (PTIs), apart from reversing cancer proton reversal also induce VEGF inhibition, so behaving as antiangiogenic drugs [20,92,107,147]. Furthermore, various anticancer drugs including adriamycin, cisplatinum, paclitaxel and camptothecin do not induce apoptosis under non-acidified intracellular conditions [148-151]. Also, resistance to several anticancer drugs such as camptothecin, vinblastine, adriamycin, and etoposide, has been correlated with overexpression of different proton transporters and/or intracellular alkalinization [4,20,83,139,140]. Besides, it should be taken into account that cytosolic acidification is a very early event in the onset of malignant cell apoptosis [106,152]. These MDR modifiers include verapamil, amiodarone, Bafilomycin A_1,_ cyclosporine A, tamoxifen, 4,4′-diisothio-cyanatostilbene-2,2′-disulfonic acid (DIDS), nigericin, cariporide and edelfosine [11].

This H^+^-based *“basic”* approach to MDR-related cancer therapeutics may lead to more selectivity and less toxicity of other chemotherapeutic agents if used together with the most potent and selective PTIs known to date, like cariporide, Phx-3 or Compound 9 t [44,81,124]. Cariporide also increases the effect of gemcitabine in human cholangiocarcinoma cells by inhibiting MDR [12]. Further along this line, the inhibition of the NHE1 has been shown to play a fundamental role in paclitaxel-induced apoptosis of breast cancer cells and this is synergistically potentiated by dimethyl amiloride (DMA) [121]. This is reasonable since this counteracts the overexpression/ activation of the NHE1 which appears to contribute to the onset and/or maintenance of MDR [5,81-83]. Thus, cariporide, because of its powerful effect in inhibiting NHE1, can also become a fundamental drug in overcoming MDR in human cancer therapy.

**MDR, proton transport inhibitors (PTI), pH and P-glycoprotein (P-gp)** In the same vein, De Milito et al. have shown that following PPI treatment of melanoma cells with esomeprazole overcomes MDR and undergo a significant decrease of proton gradient reversal, inducing tumor cell death via rapid intracellular acidification [153-155]. Also, the simultaneous inhibition of the NHE1 and H^+^-ATPase induces apoptosis through their concurrent effects on lowering pH_i_[147,156,157]. Finally, the relationships of NHE1 inhibition to tumor hypoxia, growth factors and antiangiogenic therapy have been extensively reviewed [10,74,158] and will not be further dealt with in this contribution. For detailed information on NHE/AntiNHE drug-relationships, please refer to the following original publication [107].

**Figure 4:**
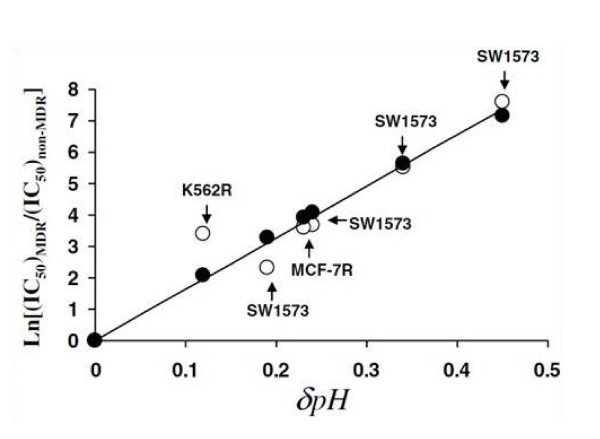
**Impact of the changes in intracellular pH on doxorubicin resistance in different cancer cells.** Multidrug resistance in cancer has been associated with the alkalization of the cytosol due to overexpression of proton pumps at the level of the cell membrane and/or expression of drug transporters. In this context it is believed that weak base drugs are protonated and as a result cannot cross the membrane bilayer, a feature that adds to the efficiency of drug transporters. Albeit this model (drug protonation and transporter) has been used over decades, the high pH of the cytosol can drive drug resistance through a different mechanism. The hypothesis made by us was that the change in cytosolic pH makes the membrane less permeable to drugs due to hydrogen-lipid interactions. To test this, a model of hydrogen-lipid interaction was formulated and compared with experimental data. In the figure the X-axis represents the positive increment in the cytosolic pH when cells switch their state from being sensitive to resistant to drugs. The Y-axis represents the ratio of the logarithm values of the concentration of drugs to kill 50% drug resistant vs. sensitive cells*.* The blank dots represent the experimental data. The black dots show the result expected from the theoretical modelling. The straight line represents the linear trend (best fit) from experimental data. Finally, the best fit passes across all the dots modelled by the theory. For further details see ref. [169].

Why is pH reversal so important in MDR? The drug handling and extrusion mechanisms mediated by P-gp glycoprotein can no longer fully account for MDR in cancer treatment [82,106,159,160]. Currently, a more integrated mechanism to explain resistance to anticancer drugs can be based upon the modification of tumor microenvironment through changes in the extracellular and intracellular pH [159,161,162]. In this regard, MDR cells exhibit a significantly high pH_i_ that accounts, at least in part, for the Pgp-mediated resistance [163] (Figure 4). The fact that cells with an active MDR transporter show such a degree of cytoplasmatic alkalinization has led some authors to conclude that P-gp can be mainly considered as a proton extrusion pump [159,161,162]. Further, P-gp activity is stimulated by the interstitial acidification of cancer tissues. Indeed, the therapeutic failure to induce cytoplasmic acidification has been proposed as the main underlying factor for MDR because it means resistance to the induction of low pH_i_-mediated *therapeutic apoptosis* in either normal, slightly alkaline and/or highly alkaline cancer cells [12,27,152,164]. This cancer antiapototic situation can be secondary to the overexpression/hyperactivity of proton transporters [76,156], the MDR-promoting effects of the Bcl-2 family of proteins [12], a dysfunctional p53 or the elevating cell pH effect of different growth factors [17,19,22,23,165,166] (see above). Incidentally, an opposite pH_i_ situation that occurs in malignancy, namely, a spontaneously occurring low pH_i_-mediated *pathological apoptosis* appears to be important in the pathogenesis of certain neurodegenerative diseases, like Alzheimer’s disease [29,30,167,168]. As we have previously considered, this suggests that the pathogenesis of cancer and certain neurodegenerative diseases can be at opposite ends of a pH-related metabolic spectrum [29]. Thus, from the point of view of apoptosis and antiapoptosis both situations are “opposite pathological processes”.

**MDR and the cell membrane** It is well known that the principal mechanism that regulates the entry of a drug into a cell is the existence of pH gradients between the extracellular environment and the intracellular compartments [170-175]. The reason why the pH gradient across the membrane is so important is related to its ability to ionize drug chemicals. While depending on the drugs, be it weak acid or weak base, it is generally agreed that ionized drugs - i.e. bearing a net positive charge due to local pH conditions, will be less prone to cross the bilayer membrane than non-ionized drugs because of their resulting ability to interact with other biological compounds. Normal tissues have a neutral extracellular/interstitial pH (pH_e_) whereas the intracellular pH (pH_i_) is slightly acidic. This should allow weakly basic drugs to enter passively into these cells. With weakly acid drugs it seems that the alkalinization of the cytosol of cancer cells can also impact on the lipid membrane by increasing the compaction of lipids making the membrane less permeable to chemicals independently of their ionization (see Figure 4) [82,161,173,176,177]. Since the increased interstitial acidity represents an advantage for the tumor to develop chemoresistance, using PTIs and/or PPIs will tend to normalize or even reverse the highly abnormal pH gradients in malignancy, leading to chemoresistance reversal.

These modifications of cancer H^+^ dynamics are associated with regression or delay of tumor growth and also with enhanced response to chemotherapy [154,177-179]. It is suggested that the environmental conditions in tumors may allow the development of new and relatively specific therapies targeting the mechanisms regulating pH_i_ under external acid conditions. Doxorubicin, cyclophosphamide, 5-fluorouracil, vincristine, vinblastine, mitoxantrone, daunorubicin and chlorambucil are all clinically useful drugs, which are ionisable and hence their distribution will be affected by the microenvironmental pH_e _[170,173-175,179]. In any case, protonation is not necessarily detrimental to drug-target interaction if the target displayed is extracellular (e.g. extracellular part of NHE1). In addition, the detrimental aspect of pH on drug-target interaction concerns weak bases only (e.g. doxorubicin). Cariporide is a weak acid (pKa ~ 4.5) and therefore protonation is beneficial as far as drug-target interaction is involved [180], and doi https://www.ebi.ac.uk/chembldb/index.php/compound/inspect/CHEMBL436559.

### On the relationship of pH_i_, pH_e_ and the NHE1 with tumor immunity

Marches et al. elegantly showed the intimate link between cancer biochemistry, molecular biology and immunity by demonstrating that the anti-IgM-mediated induction of cell death in human B lymphoma cells is dependent on NHE1 inhibition and subsequent intracellular acidification, up to a point unifying those fields under one wider embracing unit [181]. In the same vein, it is accepted that the acid component of the tumor microenvironment directly impairs the function of the anti-tumoral immune system, thus contributing to the known *in vivo* immunosuppression by hindering a “host versus graft (the grafted malignant tumor)-like reaction”. Exposure to increasingly acidic pH_e_ has also been shown to reduce tumor cell-induced cytolytic activity of lymphokine-activated killer (LAK) cells [182,183], to play a role in down-regulating cytolytic activity of tumor-infiltrating lymphocytes with natural-killer (NK) phenotype [184] and to inhibit the non-major histocompatibility complex (MHC)-restricted cytotoxicity of immunocompetent effector cells [185-188]. Most recently, it has been proposed that tumour-secreted lactic acid represents a major mechanism by which cancers can suppress the anti-cancer immune response [189]. This represents a further attempt to integrate different, so far separated fields, into larger and more all-comprehensive concepts [29], while at the same time introduces some fundamental MDR-related aspects of cancer immunity. It has also been recently shown that the NHE1, but not other isoforms, is an important mechanism in extruding H^+^ and regulating pH_i_ in immune cells themselves, such as monocytes and neutrophils, that need NHE1 to be activated to maintain an optimal pH_i_ for an effective immune defensive role [190].

From a therapeutic point of view, it has been shown that it is possible to alkalinize *in vivo* the interstitial component of malignant tumors with sodium bicarbonate or other different buffers [191] and that either acute or chronic treatment of tumor-bearing mice with sodium bicarbonate or proton pump inhibitors results in an increased antitumoral activity of different anti-tumor drugs [1]. At the present time, preliminary preclinical and clinical trials are being conducted in order to overcome the anti-immune effects of the tumoral acid extracellular component when used together with immune-stimulating measures [191,192]. A recent clinical study performed in companion animals with spontaneous tumors has shown a clear chemosensitization through a combination of high dosage proton pump inhibitors (PPIs) with different cytotoxic drugs and in tumors of different histology. This data provides a clinical proof of concept that inhibition of extracellular tumor microenvironment acidification through PPIs, PTIs and/or certain buffers may be considered a pivotal new approach in integral anticancer strategies [5,147,191-194]. This is a new and underdeveloped area that needs further research in the future.

### New and potent non amiloride-derived and non guanidine-derived compounds (SL-591227, Phx-3, compound 9 t) as promising anticancer drugs

While amiloride and some of its first synthesized derivatives were non-selective and weak NHE inhibitors [195,196], an additional series of NHE1 inhibitors whose structure is independent of amiloride have been later developed. SL-591227 was the first potent and NHE1 selective non-guanidine inhibitor [105,113,197]. The group of Tomoda developed two phenoxazine derivatives, Phx-1 and Phx-3 (for structures please see ref. [4]). Phx-3 is highly selective for NHE1 inhibition and was shown to selectively stimulate apoptosis in a variety of cancer cell lines while normal lymphocytes were not affected [44,45]. Also, PHx-3 also effectively reversed a subcutaneously injected adult T-cell leukaemia tumor growth in animal studies without noticeable toxicity (A. Tomoda, personal communication). Otherwise, researchers at Bristol-Meyers synthesized a 5-aryl-4-(4-(5-methyl-1*H*-imidazol-4-yl)piperididn-1-yl)pyrimidine analog (compound 9 t) that was reported to have an excellent NHE1 inhibitory activity (IC_50_ = 0.0065 μM), to be 500-fold more potent against NHE1 than cariporide and to have much greater selectivity for NHE1 over NHE2 (1400-fold). Besides, compound 9 has a reported 52% oral bioavailability, a plasma half-life of 1.5 hours in rats, low side-effects in mice and may possess a significantly improved safety profile over other NHE1 inhibitors [198]. Unfortunately, there have been no further publications utilizing this compound in any anticancer attempt either *in vitro* or *in vivo*.

On one hand, the development of the new non-guanidine derived NHE1 inhibitors could alleviate some of the detrimental effects of cariporide found in the EXPEDITION trial [116-118]. On the other hand, there are many reasons to think that there can be a significant selectivity of some of these NHE1 inhibitors in cancer (untested so far, at least clinically). In spite that NHE1 is ubiquitous and plays a fundamental role in pH housekeeping and volume control, it is also well known that in normal tissues the NHE1 is quiescent and is activated only during acidosis or cell shrinkage. Therefore, blocking it will have very little effect on the normal tissues. This should be an advantage to consider and exploit as an important degree of specificity in the anticancer effect of NHE1inhibitors, as it has been known from cell studies since the year 2000 [38,44].

### Towards a new and integral paradigm in human cancer therapeutics

#### Present and future prospects

The utilization of different PTIs in cancer therapeutics was originally suggested by the group of Pouysségur and our group as a novel approach to the pH-related treatment of malignant tumors because of its potential as a more selective and less toxic approach to therapeutics than conventional chemotherapy [5,71,129,199]. We conducted a preliminary clinical trial with the concerted utilization of several PTIs [5,101]. Pouysségur has also proposed the use of PTIs as a valid approach to cancer treatment, advancing that this 'pH-targeted’ therapy, perhaps combined with anti-angiogenesis in order to increase hypoxia-mediated acidosis, will synergistically induce the collapse and massive shrinkage of solid tumours [129]. Similarly, from the therapeutic point of view, reverting the Warburg effect by selective intracellular acidification has been advanced as a treatment of cancer [44]. Indeed, in the light of the older and the more recent contributions [4,28,44-46,59] it can now be concluded that counteracting the Warburg effect and its aerobic glycolysis through any therapeutic method directed to selectively induce intracellular acidification in cancer cells and/or reverting proton reversal now appears to represent one and the same phenomenon.

In summary, the most potent and promising amiloride and non-amiloride derivatives, such as cariporide, Phx-3 and compound 9 t, etc. [37,44,113,198] (see Figure 2) need to be included in pre-clinical and clinical trials as an important part of the anticancer armamentarium. That these compounds have not yet reached translational oncology becomes difficult to understand taking into account the massive theoretical background, available preclinical data as well as the results of the molecular, biochemical and metabolic studies already available at the present time. These anticancer compounds can be useful either as antitumoral and chemotherapeutic agents on their own, in the context of preventing and controlling the metastatic process and in any attempts to reverse MDR.

The effects of a targeted therapy are not durable when the therapy is designed to target a single biological molecule. This is because cellular pathways operate like webs with multiple redundancies or alternate routes that may be activated in response to the inhibition of a certain pathway. For this reason, combination and concerted therapies with PTIs will be often needed to effectively treat many tumors screened for pertinent pathway dependence. Incidentally, also in related fields like hyperthermia and radiation, it has also been known for a number of years that to keep the cytosolic pH at a certain level is fundamental as a survival mechanism, where cellular acidification increases the anticancer potential of both of these methods [187,188], alone or in combination with NHE1 inhibitors. The most potent NHE1 inhibitors could be considered alone as chemotherapeutic agents since they are able to induce intracellular acidification and/or a reverse of the abnormal proton gradient of cancer cells and tissues. It can be advanced that they show a great promise as a new and selective approach to the treatment of a wide array of different malignant tumors and even leukaemias and, hopefully, they will help to overcome the present impasse and flat progress in cancer treatment [101,200]. These strategies have been recently discussed in a review [4] and in a perspective [5] and introduce a real paradigm shift in cancer treatment.

## Conclusions

1) Cell acid–base balance is recognized to be the main parameter to define cellular homeostasis, the life of cells being possible only within a very narrow range of pH (less that one unit). In that context, the pH of normal cells and cancer cells deviate towards opposite ends of a biological and metabolic spectrum. This energetic abnormality represents the largest difference among normal cellular physiology and cancer pathophysiology.

2) From an etiological and ethiopathogenic perpective, the hydrogen-related dynamics of malignancy have become a new approach to cancer that is helping to reach a better understanding of several, until now disparaged areas of cancer research both at basic and clinical levels, as well as of the intimate nature of the malignant disease. This unifying thermodynamic view permits an integration of different cancer fields, ranging from cell transformation and metabolism, local growth and invasion to neovascularisation and the activation and progression of the metastatic process (pH centric paradigm).

3) From a therapeutic perspective, the primary aim of this pH-based approach to cancer treatment is to manipulate the selective forces controlling the dysregulated pH dynamics of all cancer cells and tissues in order to regress tumor growth, control local invasion and deactivate the metastatic potential of malignant tumors within the same integral perspective and paradigm. All available evidence seems to indicate that this would take place regardless of pathological differences, tissue type or genetic origin. This therapeutic approach would also provide much less toxicity than present day treatments, probably more effective therapies than any other chemotherapy known to date and it has real possibilities to become a successful strategy in treating human cancer in general. A pathologically elevated pH_i_ and its associated proton reversal (a reversed pH gradient in cancer cells and tissues (∆pH_i_ to ∆pH_e_, ↑pH_i_/↓pH_e_) can be now considered a most specific cancer abnormality and essential hallmark of all kinds of malignant cells and tissues.

4) It can be concluded that aerobic glycolysis or damaged respiration was not the primary cause of cancer, as Warburg incorrectly defended until his death. It now seems more likely that the primary cause of cancer is, precisely, the main cause of the aerobic glycolysis of tumors. And this is that the abnormally high intracellular pH of cancer cells, mediated by a myriad of etiological factors of many different natures, can very well be the real cause of cancer. Furthermore, this tendency towards cellular alkalinity appears to be an specific and selective characteristic of cancer since it has not been described in any other disease.

5) This hydrogen ion-based perspective has also permitted the better understanding of the Warburg effect, which can now be simply explained by the effects of the concerted action of proton transporters in increasing intracellular pH and stimulating aerobic glycolysis. In this respect, Otto Warburg and his contemporaries committed an important historical error that has possibly misled several decades of metabolic and biochemical cancer research. The main limitation was probably imposed by the lack of available intracellular pH measurements before the time of Warburg’s death in 1970. The high pH_i_ of tumor cells, the Warburg effect and the proton reversal of cancer cells and tissues are likely to represent one and the same phenomenon defined in different ways.

6) Many different environmental and chemical carcinogens have been shown to be cancer-inducing agents because of their potential to stimulate NHE1 activity with the subsequent increase in intracellular pH and decrease in microenvironmental pH. This cancer-inducing mechanism opens an entire new area in cancer epidemiology looking for generalizations both in detecting and controlling environmental carcinogens.

7) Any attempt to therapeutically induce a selective intracellular acidification using proton transport inhibitors (PTIs) in all cancer cells and tissues would secondarily increase interstitial tumoral pH, thus inhibiting the metastatic process, and represents a rational and firmly based approach to cancer treatment in all stages of development. Further, it has the potential of being selectively exploited in the treatment of many different malignant solid tumours.

8) Cariporide, other potent NHE1 inhibitors of the amiloride series, as well as powerful and selective NHE1 inhibitors of the non-amiloride series, like Phx-3 and compound 9 t, have the potential of being highly promising, minimally toxic and truly effective anticancer agents in a wide array of malignant tumours and leukaemias, hopefully representing a new paradigm in cancer therapeutics.

## Competing interests

The authors declare that the research was conducted in the absence of any commercial or financial relationships that could be or become a potential conflict of interests.

## Authors’ contributions

SH conceived the review, participated in its design, wrote the first drafts and collaborated in writing and correcting most sections. SJR conceived the review, participated in its design and collaborated in writing and correcting some sections. RAC helped to correct parts of the sections on the role of the NHE1 in oncogenesis and on anticancer potential of NHE inhibitors. JLA and JPO participated in the general script of the review as well as collaborated in the work explained in different figures and tables as well as in collecting and selecting at least half of the list of references. JLA actively participated in discussing and forwarding ideas on the etiology of the Warburg effect. JPO helped to criticize parts of the section on the anticancer potential of NHE inhibitors. CR main contribution was writing on pH, MDR and cancer and pH and the cell membrane. SF wrote parts of the section on MDR, proton transport inhibitors (PPI), pH and P-gp., and shared his ideas and experience on the adjuvant role of PPIs treatment in animal and human cancer. All authors read and approved the final manuscript.
